# 

**Published:** 1891-08

**Authors:** 


					﻿ESTABLISHED 1855
SUBSCRIPTION, $2.50.

MEDIS
WML
uDURNAU
EDITED BY
H. V. M. MILLER, M. I)., LL. I).
VIRGIL O. IJARDON, M. D.
F. W. McRAE, M. D.
L.	P. KENNEDY, A. M„ M. D.
M.	B. HUTCHINS, M. D.
L. B. GRANDY, M. D.
Published by JAS. P. HARRISON A < O., Atlanta, Ha.
Entered at the Post-’ tffice at Atlanta, Ga.. as second-class matter.
-- — --------■
Newskriks Vm" Vu\ I ATLANTA, Ga., AUGUST, 1891^	No. 6.
				

